# ASO Author Reflections: Safety of Performing Esophageal Cancer Surgery During the First Wave of the COVID-19 Pandemic in Europe: A Multicenter Study

**DOI:** 10.1245/s10434-021-09891-2

**Published:** 2021-03-24

**Authors:** Alexander B. J. Borgstein, Mark I. van Berge Henegouwen

**Affiliations:** Department of Surgery, Amsterdam UMC, University of Amsterdam, Cancer Center Amsterdam, Amsterdam, The Netherlands

## PAST

Many centers in the world postponed elective surgical care during the first wave of the coronavirus disease 2019 (COVID-19) pandemic due to a number of factors. First, hospitals had to shift medical resources to increase intensive care unit (ICU) capacities and other wards to treat increasing numbers of COVID-19 patients, and second, to prevent patients and healthcare workers from potentially acquiring in-hospital severe acute respiratory syndrome coronavirus 2 (SARS-CoV-2) infections. This strategy to postpone elective surgery was supported by a large study indicating patients with a SARS-CoV-2 infection undergoing surgery had an increased risk for postoperative pulmonary complications and mortality.^1^ Many centers were forced to postpone treatment for esophageal cancer patients for a long period of time. Moreover, some centers switched to alternative treatment measures, including definite chemoradiotherapy.

On the other hand, some centers were able to continue elective cancer surgery, including esophageal cancer surgery, with the use of effective preoperative screening methods.^2^ However, there was no evidence supporting the safety of continuing elective esophageal cancer surgery during the first wave of the COVID-19 pandemic. Additionally, several societies released guidelines in which the use of minimally invasive surgery was discouraged, as these procedures could potentially contaminate surgical staff with SARS-CoV-2.

## Present

The current international, multicenter cohort study assessed the safety of continuing esophageal cancer surgery by comparing the rate of respiratory failure requiring mechanical ventilation between a COVID-19 cohort and a control cohort of patients undergoing elective esophageal cancer surgery.^3^ The rate of respiratory failure requiring mechanical ventilation was comparable between both cohorts (13.7% in the COVID-19 cohort vs. 8.3% in the control cohort), as was the number of pulmonary complications (32.4% vs. 29.9%). Additionally, there was no difference in the overall 30-day mortality rate between both cohorts. Preoperative history taking and reverse transcriptase polymerase chain reaction (RT-PCR) testing were used in all participating centers as screening methods and no patients tested positive for COVID-19 pre- or postoperatively. Seventy-five percent of all esophagectomies were performed minimally invasive. Our study did not assess the COVID-19 presence among surgical staff; however, as no patients were diagnosed with COVID-19 pre- or postoperatively, minimally invasive surgery could be used safely.

The results of the current study provide evidence for the safety of continuing elective esophageal cancer surgery, and potentially all other major cancer surgeries, during the ongoing COVID-19 pandemic under the condition that a secure screening protocol is in place for patients undergoing esophageal cancer surgery.

## Future

The study concluded that, globally, up to 72% of all adult elective surgeries were cancelled during the first 12 weeks of the COVID-19 pandemic.^4^ Additionally, almost 38% of all cancer surgeries have been postponed. To date, no studies have been conducted indicating tumor progression or worse overall survival because of postponement of cancer surgery; however, one might expect this relationship to be present. Recently, a study analyzed the progression of gastrointestinal tumors in patients presenting during the COVID-19 pandemic. Patients with gastric cancer underwent less surgery and were diagnosed with more lymph node (pN+) and distant metastases (cM1).^5^ That study indicates the importance of continuing cancer surgery and our study provides the evidence that it can be performed safely with adequate preoperative screening methods.

Currently, most countries worldwide are going through a second COVID-19 wave and some are even facing a potential third wave. These new waves are characterized by the introduction of new SARS-CoV-2 variants, which have an even higher transmissibility. Therefore, hospitals may face increased numbers of COVID-19 patients in the coming months, which will affect surgical and ICU capacities. However, with the use of patient selection and adequate preoperative screening methods, elective cancer surgery can be continued. Tumor progression because of postponement of surgical care or the use of less effective alternative treatment options could be prevented.
